# Hotspots and trends in fNIRS disease research: A bibliometric analysis

**DOI:** 10.3389/fnins.2023.1097002

**Published:** 2023-03-02

**Authors:** Xiangyin Ye, Li Peng, Ning Sun, Lian He, Xiuqiong Yang, Yuanfang Zhou, Jian Xiong, Yuquan Shen, Ruirui Sun, Fanrong Liang

**Affiliations:** ^1^Acupuncture and Tuina School, Chengdu University of Traditional Chinese Medicine, Chengdu, China; ^2^Department of Ultrasound, The First People’s Hospital of Longquanyi District, Chengdu, China; ^3^Rehabilitation Medicine Center and Institute of Rehabilitation Medicine, West China Hospital, Sichuan University, Chengdu, China; ^4^Department of Rehabilitation Medicine, The First People’s Hospital of Longquanyi District, Chengdu, China

**Keywords:** functional near-infrared spectroscopy (fNIRS), CiteSpace, diseases, Frontiers, bibliometrics

## Abstract

**Objective:**

To summarize the general information and hotspots of functional near-infrared spectroscopy (fNIRS)-based clinical disease research over the past 10 years and provide some references for future research.

**Methods:**

The related literature published between 1 January 2011 and 31 January 2022 was retrieved from the Web of Science core database (WoS). Bibliometric visualization analysis of countries/regions, institutions, authors, journals, keywords and references were conducted by using CiteSpace 6.1.R3.

**Results:**

A total of 467 articles were included, and the annual number of articles published over nearly a decade showed an upward trend year-by-year. These articles mainly come from 39 countries/regions and 280 institutions. The representative country and institution were the USA and the University of Tubingen. We identified 266 authors, among which Andreas J Fallgatter and Ann-Christine Ehlis were the influential authors. Neuroimage was the most co-cited journal. The major topics in fNIRS disease research included activation, prefrontal cortex, working memory, cortex, and functional magnetic resonance imaging (fMRI). In recent years, the Frontier topics were executive function, functional connectivity, performance, diagnosis, Alzheimer’s disease, children, and adolescents. Based on the burst of co-cited references, gait research has received much attention.

**Conclusion:**

This study conducted a comprehensive, objective, and visual analysis of publications, and revealed the status of relevant studies, hot topics, and trends concerning fNIRS disease research from 2011 to 2022. It is hoped that this work would help researchers to identify new perspectives on potential collaborators, important topics, and research Frontiers.

## 1. Introduction

Functional near-infrared spectroscopy (fNIRS) is an optical neuroimaging technique used to quantify oxy- and deoxyhemoglobin content for functional brain imaging (Irani et al., 2007). As an emerging technology, fNIRS has played an increasingly important role in functional neuroimaging over the past 10 years (Ferrari and Quaresima, 2012; Rahman et al., 2020). In addition to its non-invasiveness, radiation-free nature, wearability and portability, and insensitivity to motion artifacts, fNIRS also provides higher temporal/spatial resolution than functional magnetic resonance imaging (fMRI) and electroencephalography (EEG). These advantages make fNIRS eminently suitable for application in special cohorts such as infants (Doi and Shinohara, 2017; McDonald and Perdue, 2018), patients with motor impairments (Gramigna et al., 2017; Ranchet et al., 2020), and patients with neurological conditions (Koike et al., 2013; Kumar et al., 2017).

Functional near-infrared spectroscopy has recently developed rapidly in conjunction with advancements in near-infrared spectroscopy (NIRS) hardware, applications, and data analysis methods (Pinti et al., 2020; Eastmond et al., 2022). It has been widely used for brain function assessments under various neuropsychiatric conditions, including, but not limited to, attention-deficit/hyperactivity disorder (ADHD), affective disorders, neuropathic pain, and cognitive impairment (Ehlis et al., 2014; Kumar et al., 2017; Yeung and Chan, 2020). Meanwhile, multimodal neuroimaging studies that include fNIRS are ideally suited to examine brain function. For example, EEG-fNIRS examinations can be used to identify more features correlated with brain activation and connectivity (Muthalib et al., 2013).

With the increase in fNIRS research, several scholars have summarized the research on the application of fNIRS to a specific disease or disease system (Koike et al., 2013; Doi and Shinohara, 2017; Kumar et al., 2017; McDonald and Perdue, 2018; Pinti et al., 2020). However, the overall application of fNIRS technology in assessing the brain imaging characteristics of disease activity has not been summarized. Determination of the global research trends and hotspots of fNIRS research on clinical diseases is important to provide references for future studies.

Therefore, in this study, CiteSpace was used to conduct a thorough bibliometric analysis of research related to the use of fNIRS for clinical diseases from 2011 to 2022. The purpose of our study was to provide scholars who have recently entered the field and others who will soon follow with new perspectives on the status of relevant studies, important topics, and trends concerning fNIRS in clinical disease research from a global perspective.

## 2. Materials and methods

### 2.1. Source and retrieval

In this study, Web of Science (WoS) was selected as the data source, and the “advanced search” method was adopted. The search formula was “TS = (fNIRS or functional near-infrared spectrum or near-infrared spectrum).” Literature published from 1 January 2011 to 31 January 2022 was searched, and only English language documents were eligible for subsequent analyses.

### 2.2. Inclusion and exclusion criteria

We limited this analysis to studies involving patients (adult patients or children). The types of studies included clinical trials and efficacy observation studies with more than two cases. Non-medical research papers, papers with animals/healthy individuals as research objects, technical exploration papers, degree papers, and case reports, etc., were excluded.

### 2.3. Screening method

Two reviewers independently screened the articles by evaluating the titles and abstracts on the basis of the inclusion criteria described above. The full text of the articles was reviewed if necessary. Disagreements were discussed and resolved by the two reviewers, and further disputes were arbitrated by a third reviewer.

**FIGURE 1:**
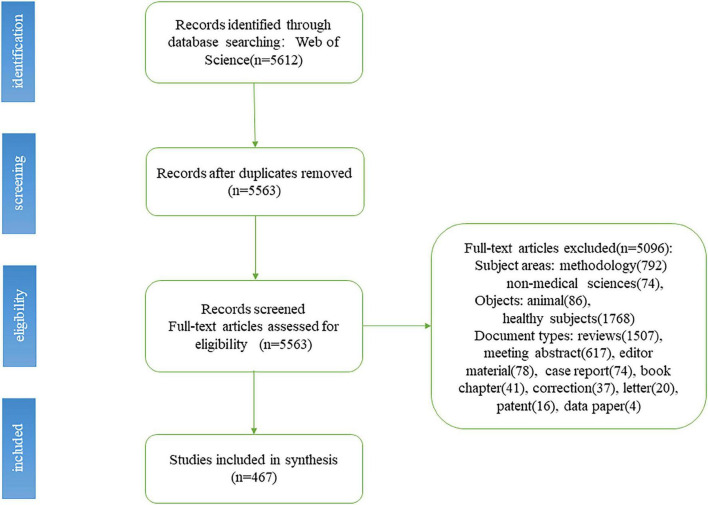
Flow chart of functional near-infrared spectroscopy (fNIRS) studies inclusion.

A total of 5,612 related studies were retrieved from the WoS core database. After screening the literature in accordance with the criteria described above, 467 studies were finally included. The specific process is shown in Figure 1.

### 2.4. Data acquisition and analytical tools

This study used CiteSpace 6.1.R3 for analysis by combining the literature metrology method, visual analysis method, and data mining algorithm. The relevant documents were manually screened and exported in the pure text format with the name “download_xx.txt.” Duplicate records were removed in CiteSpace and stored in the data folder. The exported information included data regarding the author, title, keywords, abstract, source publication, document type, citation frequency, highly cited papers, research direction, and page number.

In the visual diagram, each node represents an element, and the size of the node represents the frequency of occurrence. The connection line represents the cooperation or connection between the elements. Nodes showing high betweenness centrality (> 0.1) are usually considered to be key points in the field. CiteSpace highlights nodes with high betweenness centrality using purple trims. Highly cited elements are often considered to be research hotspots in that particular field. Three special indicators–latent semantic indexing (LSI), log-likelihood test (LLR), and mutual information test (MI)–were used in the cluster analysis, and we mainly observed the outcomes of LLR, which provided the best result in terms of themes associated with a cluster. The CiteSpace parameters were as follows: time-slicing, from January 2011 to January 2022 (1 year per slice); term source, all selection; node type, choose one at a time; and pruning, pathfinder.

## 3. Results

### 3.1. Annual publication trends

**FIGURE 2:**
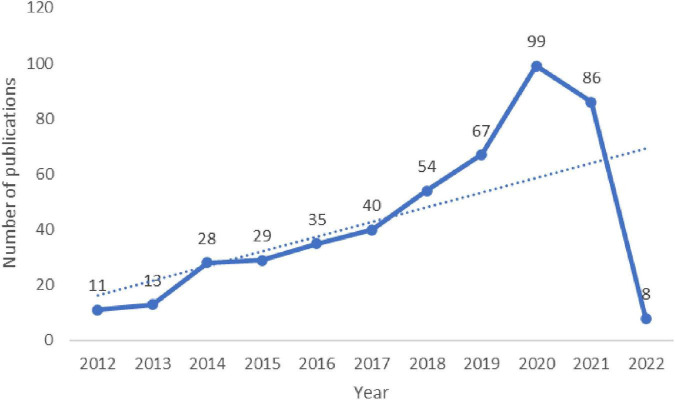
Annual publication outputs and the model fitting curve of time trend of relevant publications.

A total of 467 publications were included. From 1 January 2011 to 31 January 2022, the total number of studies involving fNIRS for disease research gradually increased and reached the highest value in 2019. Although the included literature from 2022 did not represent the complete year, the overall trend was still upward (Figure 2), and since 2018, the total number of articles published each year has exceeded 50.

### 3.2. Analysis by country/region

**FIGURE 3:**
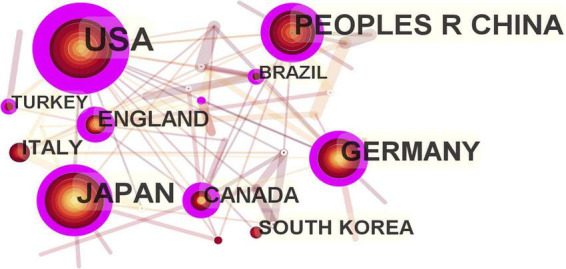
Map of active country in functional near-infrared spectroscopy (fNIRS) research on diseases.

The maps drawn in the national visual analysis included 39 countries and 82 connection lines. The top three countries in terms of the number of articles published were the USA, China, and Japan (Figure 3). The number of articles published by authors from these countries was 134, 83, and 70, respectively. Notably, only China (ranked 2nd) entered the top 10 as a developing country, while the remaining nations are all developed countries. Purple rings indicated countries with high centrality (≥ 0.1). The top five countries in terms of centrality were USA (0.74), Germany (0.39), China (0.34), England (0.32), and Canada (0.27). These five countries were influential in the field of fNIRS research.

### 3.3. Analysis by institution

**FIGURE 4:**
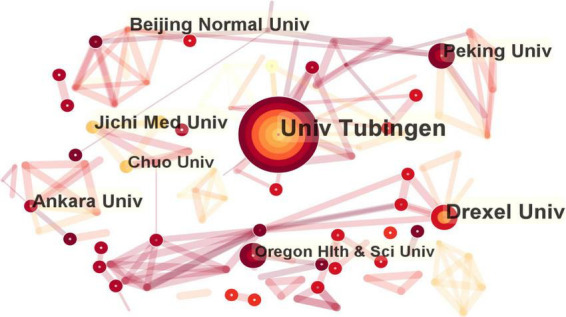
Map of active institution in functional near-infrared spectroscopy (fNIRS) research on diseases.

As shown in the institutional visualization map, 280 institutions contributed to the literature on fNIRS in disease research. Among these, six institutions had contributed more than 10 studies (frequency in brackets): University of Tubingen (26), Drexel University (15), Jichi Medical University (11), Ankara University (11), Beijing Normal University (10), and Peking University (10), as shown in Figure 4. Most of the contributing institutions were universities.

### 3.4. Analysis by authors

**FIGURE 5:**
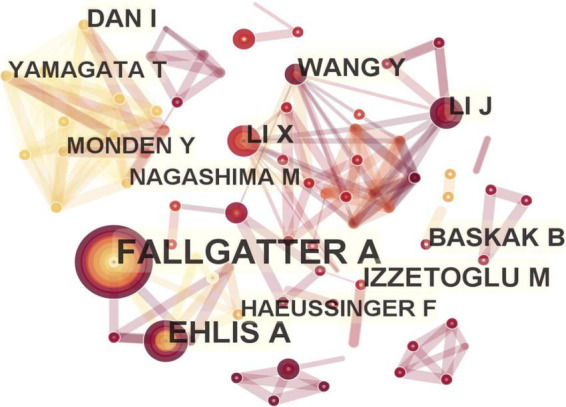
Map of active authors in functional near-infrared spectroscopy (fNIRS) research on diseases.

The number of nodes in the author visualization map was 266, and the number of connections was 854. The following eight authors had published more than 10 papers, and they represented the major researchers and teams conducting in-depth research with fNIRS from 2011 to 2022 (frequency in brackets): Andreas J Fallgatter (28) and Ann-Christine Ehlis (22) from University of Tubingen, Meltem Izzetoglu (14) from Drexel University, Yuduo Wang (11) from Beijing Information Science and Technology University, Xiaoli Li (11) from Beijing Normal University, Ippeita Dan (11) from Chuo University, Bora Baskak (10) from Ankara University, and Jun Li (10) from South China Normal University (Figure 5).

### 3.5. Analysis of the journals and cited journals

**FIGURE 6:**
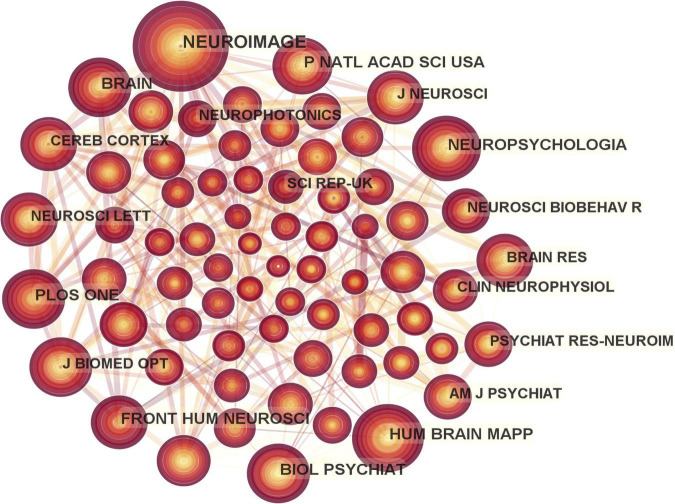
Map of cited journals in functional near-infrared spectroscopy (fNIRS) research on diseases.

A total of 175 journals published papers on the application of fNIRS in disease, Table 1 shows the top 10 academic journals that published related papers, with the average impact factor (IF) 4.251 (median 4.37; range 3.333–4.997). The journals with more than 20 publications are Scientific Reports and Frontiers in Human Neuroscience. In this study, a map of 432 nodes and 1,868 connecting lines was formed after visual analysis of the cited journals (Figure 6). For the top 10 cited journals shown in Table 2, the average IF was 7.439 (median 6.054; range 3.054–15.255). The top five cited journals were Neuroimage, PLoS One, Human Brain Mapping, Frontiers in Human Neuroscience, and Brain. Except for PLoS One, the other four journals were brain imaging journals.

**TABLE 1:** The top 10 journals for functional near-infrared spectroscopy (fNIRS) research on diseases.

Rank	Journal	Frequency	Country	IF (2021)*	CiteScore
1	Scientific Reports	24	England	4.997	6.9
2	Frontiers in Human Neuroscience	23	Switzerland	3.473	4.6
3	Neuroimage-clinical	18	Netherlands	4.891	8.2
4	Neurophotonics	17	USA	4.212	8.3
5	Neurorehabilitation and Neural Repair	14	USA	4.895	6.1
6	PLoS One	13	USA	3.752	5.6
7	Brain Sciences	12	Switzerland	3.333	3.1
8	IEEE Transactions on Neural Systems and Rehabilitation Engineering	11	USA	4.528	8.1
9	Schizophrenia Research	9	Netherlands	4.662	8
10	Biomedical Optics Express	9	USA	3.562	6.7

*IF, impact factor; IF in category according to journal citation reports (2021).

**TABLE 2:** The top 10 co-cited journals for functional near-infrared spectroscopy (fNIRS) research on diseases.

Rank	Journal	Frequency	Country	IF (2021)	CiteScore
1	Neuroimage	402	USA	7.400	11.2
2	PLoS One	235	USA	3.752	5.6
3	Human Brain Mapping	210	USA	5.399	8.3
4	Frontiers in Human Neuroscience	194	Switzerland	3.473	4.6
5	Brain	172	England	15.255	19.7
6	Biological Psychiatry	150	USA	12.810	21.5
7	Neuropsychologia	150	England	3.054	5.8
8	Proceedings of the National Academy of Sciences	149	USA	12.778	18.1
9	Journal of Neuroscience	138	USA	6.709	10.2
10	Journal of Biomedical Optics	135	USA	3.758	6.6

### 3.6. Analysis of keywords

**FIGURE 7:**
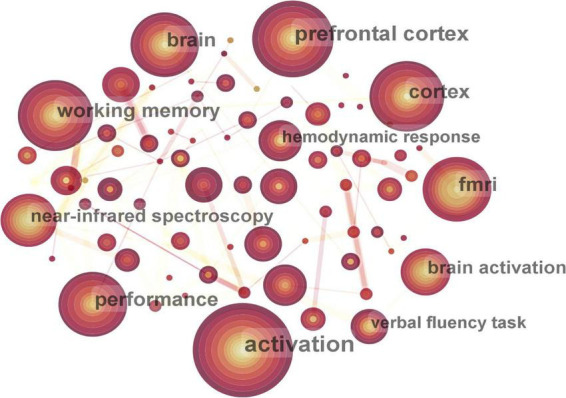
Map of active keywords in functional near-infrared spectroscopy (fNIRS) research on diseases.

The keyword co-occurrence map included 343 nodes and 1,209 connecting lines (Figure 7). Nine keywords occurred more than 50 times. Excluding the words related to the retrieval strategy, the top seven keywords were activation, prefrontal cortex (PFC), working memory, cortex, fMRI, performance, and brain. Keywords with high centrality were brain (0.18), cortex (0.15), abnormality (0.12), and PFC (0.1).

Fifty-four cluster labels were obtained by using LLR algorithm. The mean silhouette was 0.9578, with good homogeneity and reliable results. The top 10 clusters were #0 verbal fluency task, #1 graph theory, #2 concussion, #3 gait, #4 optical topography, #5 stimulus-specific adaptation, #6 primary motor cortex, #7 motor control, #8 cue reactivity, and #9 multimodal neuroimaging. The main clusters and their keywords are shown in Table 3.

**TABLE 3:** The list of main keyword clusters.

Cluster ID	Size	Silhouette	Mean (year)	Top terms (LSI)*	Top terms (LLR)*	Terms (MI)*
0	76	0.909	2015	Prefrontal cortex	Verbal fluency task	Percussion
1	74	0.906	2016	Functional near-infrared spectroscopy	Graph theory	Discrimination
2	74	0.963	2014	Activation	Concussion	Motor inhibition
3	71	0.905	2015	Prefrontal cortex	Gait	Cerebral oxygenation
4	64	0.951	2015	Optical topography	Optical topography	Inhibitory task-evoked activation
5	59	0.975	2016	Functional near-infrared spectroscopy	Stimulus-specific adaptation	Stimulus-specific adaptation
6	55	0.952	2016	Functional near-infrared spectroscopy	Primary motor cortex	Upper limb reduction
7	55	0.984	2015	Motor control	Motor control	Brain map
8	51	0.978	2016	Optical topography	Cue reactivity	Cue exposure
9	51	0.947	2017	Functional near-infrared spectroscopy	Multimodal neuroimaging	Brain computer interface

*LSI, latent semantic indexing; LLR, log-likelihood test; MI, mutual information test.

The keyword burst analysis indicated 14 outbreak points, as shown in Table 4. The points with high outbreak intensity were as follows: Executive function (2017–2021), optical topography (2012–2015), and functional connectivity (2017–2021). These points represent important aspects in the field of disease research with fNIRS. Research burst is considered to be continuous. Topics such as executive function, functional connectivity, performance, diagnosis, Alzheimer’s disease, children, and adolescents have the potential to continue to become research hotspots in the near future, and represent research content and topics worthy of special attention.

**TABLE 4:** Top 12 keywords with the strongest citation bursts.

Keywords	Year	Strength	Begin	End	2011–2021
Optical topography	2011	3.94	2012	2015	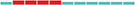
Near-infrared spectroscopy	2011	3.55	2012	2013	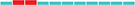
Blood flow	2011	3	2013	2014	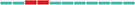
fMRI	2011	2.99	2014	2015	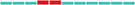
Plasticity	2011	3.07	2017	2018	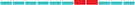
Executive function	2011	5.09	2019	2021	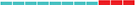
Functional connectivity	2011	3.92	2019	2021	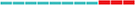
Performance	2011	3.67	2019	2021	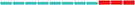
Diagnosis	2011	3	2019	2021	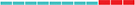
Alzheimer’s disease	2011	2.99	2019	2021	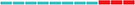
Children	2011	2.99	2019	2021	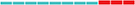
Adolescent	2011	2.77	2019	2021	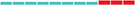

### 3.7. Analysis of references

Visual analysis of co-cited references was performed in CiteSpace, and a map with 4,072 nodes and 14,308 connections was generated. The top five co-cited references are listed in Table 5, which are five reviews that introduce the instruments and methods in near-infrared spectral imaging technology (Ehlis et al., 2014), the history of development and application fields of fNIRS (Ferrari and Quaresima, 2012), the applications of fNIRS in psychiatry (Scholkmann et al., 2014), the functional research progress of fNIRS in the last 20 years (Boas et al., 2014), and evidence-based suggestions for fNIRS walking research design and signal analysis technology (Vitorio et al., 2017).

**TABLE 5:** Top five cited references related to functional near-infrared spectroscopy (fNIRS) research on diseases.

Ranking	Co-cited counts	Co-cited reference	References
1	29	Application of functional near-infrared spectroscopy in psychiatry.	Ehlis et al., 2014
2	29	A brief review on the history of human functional near-infrared spectroscopy (fNIRS) development and fields of application.	Ferrari and Quaresima, 2012
3	29	A review on continuous wave functional near-infrared spectroscopy and imaging instrumentation and methodology.	Scholkmann et al., 2014
4	26	Twenty years of functional near-infrared spectroscopy: introduction for the special issue.	Boas et al., 2014
5	18	fNIRS response during walking—artifact or cortical activity? A systematic review.	Vitorio et al., 2017

The burst citations of references indicate numerous citations of the manuscript over a certain period of time. Table 6 shows the 11 studies with the strongest reference bursts from 2011 to 2021. The strongest reference burst was recorded for a review of the development history and application fields of fNIRS in humans published by Ferrari and Quaresima (2012). A more recent study with a reference burst was a paper published by Nieuwhof et al. (2016).

**TABLE 6:** Top 11 co-cited references with the strongest citation bursts.

References	Year	Strength	Begin	End	2011–2021
Schecklmann et al., 2008	2008	5.9	2011	2013	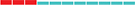
Schecklmann et al., 2010	2010	3.27	2011	2013	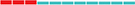
Negoro et al., 2010	2010	3.17	2012	2014	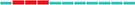
Rubia et al., 2009	2009	2.54	2012	2014	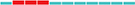
Ferrari and Quaresima, 2012	2012	9.23	2013	2017	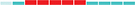
Cui et al., 2011	2011	4.68	2013	2016	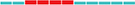
Holtzer et al., 2011	2011	3.27	2013	2016	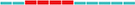
Haeussinger et al., 2011	2011	4.93	2014	2016	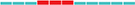
Sasai et al., 2011	2011	3.45	2014	2016	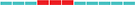
Kirilina et al., 2012	2012	2.73	2014	2017	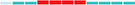
Nieuwhof et al., 2016	2016	2.9	2018	2021	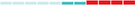

## 4. Discussion

As a non-invasive brain functional imaging technique, fNIRS plays an increasingly important role in the detection of function-related activity. The current study applied a visualization-based bibliometric method to analyze the profiles, research hotspots, and research trends in relation to the use of fNIRS technology in disease research. fNIRS is gradually becoming an important technique for the studying related diseases in the field of neuroimaging.

### 4.1. General information regarding fNIRS research on diseases

In terms of the country-wise distribution of the published literature on this topic, the USA ranks first in the number and centrality of literature, indicating its leading position in this research field. The brain program implemented by the National Institutes of Health (NIH) in the USA since 2013 has provided huge financial support and driven advancements in clinical research in neuroimaging. The major institutions contributing to the research output in this topic were University of Tubingen in Germany, Drexel University in USA, and Jichi Medical University in Japan, which was consistent with the analysis results of national distribution. Among the most prolific authors, Andreas J Fallgatter and Ann-Christine Ehlis published as many as 16 manuscripts, mainly focusing on ADHD and depression (Ehlis et al., 2007; Zeller et al., 2010; Marx et al., 2014; Metzger et al., 2016a,b). However, the findings indicate that institutional and author cooperations are lacking and need to be strengthened. In addition to the emphasis on neuroimaging, the research direction of journals indicates that most of the publications involve neuroscience, psychiatry, and multidisciplinary sciences. Further collaboration should be implemented in the future to obtain more significant evidence.

### 4.2. Research hotspots and trends in fNIRS research on diseases

Disease research using fNIRS was more focused on the activation of brain regions of patients in the task state, with an emphasis on the PFC. The typical activation of fNIRS in observed in the cerebral cortex area, that is, the increase in oxygenated hemoglobin (HbO) and the relative decrease in deoxygenated hemoglobin (HHb) reflect the increase in local cerebral blood capacity caused by increased local arterial vascular relaxation. The PFC is closely related to cognitive functions such as working memory, stimulus selection, rule switching, and decision-making (Ott and Nieder, 2019), and its dysfunction is associated with social deficits, affective disturbances and memory loss in brain disorders including autism, schizophrenia, depression, and Alzheimer’s disease (AD) (Yan and Rein, 2022). For example, Krishnamurthy et al. found that children with autism had altered PFC function, manifested by PFC hyperactivation and decreased right frontal connectivity (Krishnamurthy et al., 2020). Xiang et al. found that the activation of the extensive PFC was significantly reduced in patients with schizophrenia and major depressive disorder (MDD), especially in certain channels of the dorsolateral PFC (DLPFC) (Xiang et al., 2021). Tang and Chan found that the functional connectivity of PFC in patients with AD generally decreased, the laterality became insignificant, and the clustering coefficient decreased significantly (Tang and Chan, 2018). Working memory is a memory system that temporarily processes and stores information and is an important basis for complex cognitive tasks (Baddeley, 1992). The PFC is thought to play a key role in encoding, updating, and maintaining internal representations of task situations in working memory (D’Ardenne et al., 2012). fNIRS can quantify the workload of PFC during experimental conditions (Herff et al., 2013), which is helpful for the neuropsychological assessment of working memory under n-back tasks (León-Domínguez et al., 2015). The “Verbal Fluency Task (VFT),” which showed the largest cluster in keyword clustering, is a neuropsychological task involving multiple cognitive domains, which can reflect the fluency of individuals in using language to transmit information (Liu et al., 2022). The combined monitoring of fNIRS and VFT provides neural evidence of executive function in patients and is widely used in the diagnosis and differential diagnosis of depression, bipolar disorder, and schizophrenia (Koike et al., 2013; Downey et al., 2019; Feng et al., 2021; Xiang et al., 2021; Gao et al., 2022). The primary motor cortex (M1) is involved in the control of advanced gait movements (McCrimmon et al., 2018). When the function of the motor cortex changes due to brain injury, stroke, Parkinson’s disease and other diseases, it may lead to abnormal gait. Clinically, regulating the activity of M1 may be an effective treatment to improve the motor function of patients (Underwood and Parr-Brownlie, 2021). In addition, motor control, defined as the ability to regulate or manage the mechanisms necessary for movement (Levin and Piscitelli, 2022), is an effective rehabilitation strategy for abnormal gait after stroke and is an important theory in clinical rehabilitation at present (Beyaert et al., 2015). fMRI is the gold standard for functional imaging of the brain, but it suffers from some shortcomings in terms of temporal resolution. The sampling rate of fNIRS can reach 0.1 s, and at the same time, there is a strong correlation between the hemoglobin signal of fNIRS and the BOLD signal of fMRI (Strangman et al., 2002). Multimodal neuroimaging can complement each other and provide more information.

Keyword bursts indicate emerging trends and potentially valuable research directions to a certain extent. In this study, executive function, functional connectivity, performance, diagnosis, AD, children, and adolescent were the most recent burst keywords. The relevance of these keywords can be summarized as follows: (1) Executive function, including working memory, cognitive flexibility and inhibition, is closely related to PFC function (Jones and Graff-Radford, 2021). Executive dysfunction is extremely common in neurological diseases (Rabinovici et al., 2015). fNIRS studies of ADHD showed hypoactivity in the right PFC in multiple executive function tasks, which was essentially consistent with the results of fMRI (Gossé et al., 2022). (2) Functional connectivity refers to the information flow between brain networks to explore the interactions between different brain functional areas, which is an important area of disease research, especially for diseases considered to be related to connectivity. In this regard, narrowband resting-state functional connectivity based on fNIRS measurements can be used for prediction of autism spectrum disorder (Sun et al., 2021). (3) By tracking the progression of working memory tasks, fNIRS revealed a link between brain activity and task performance (Meidenbauer et al., 2021). During cooperative tasks, better task performance was associated with interpersonal brain synchronization (Zhou et al., 2022). Patients with schizophrenia (SZ) performed worse on the VFT than patients with MDD and healthy controls, and patients performed worse on the Tower of London task than healthy controls (Xiang et al., 2021). (4) In terms of disease diagnosis and identification, Zhu et al. studied the brain function of autistic patients during related tasks through fNIRS (Zhu et al., 2015; Li and Yu, 2016; Vo et al., 2021); Li et al. found that the HbO concentration in the region of interest changed sharply as the disease severity progressed from mild cognitive impairment to moderate/severe dementia (Li et al., 2018; Nakamura et al., 2021); Stuart et al. measured the activity of the PFC during a task by using fNIRS to distinguish patients with Parkinson’s disease and healthy individuals (Stuart et al., 2019); and Chou et al. used fNIRS to measure the correlation between the activation of specific brain regions in patients with schizophrenia and their clinical symptoms and functional results (Chou et al., 2020), facilitating clinical differentiation of schizophrenia, depression and bipolar disorder (Kumar et al., 2017). (5) fNIRS is also of great significance in the early diagnosis of AD (Perpetuini et al., 2019), since it can distinguish AD from normal aging based on functional connectivity (Tang and Chan, 2018), and can be used for AD screening with the help of refined PFC working memory-related networks (Kim et al., 2021). fNIRS revealed different patterns of activation in the frontoparietal cortex between AD and behavioral-subtype frontotemporal dementia (Metzger et al., 2016b), and showed more severe disruption of connectivity and frontal oxygenation changes in AD patients than in patients with mild cognitive impairment patients (Yeung and Chan, 2020), which can facilitate AD diagnosis and identification of disease progression. (6) Because of its high ecological efficiency, fNIRS can be used to conduct functional neuroimaging research in a participant-friendly environment (Chou et al., 2020). Moreover, fNIRS also shows a high tolerance for the influence of patients’ head movements. Thus, it is particularly useful in research on infants and adolescents, and is widely used in the studies of ADHD, Parkinson’s disease, epilepsy, etc.

Our analysis of the cited references shows that continuous advancements and improvements in fNIRS instruments and technical methods will provide strong conditions for expanding the field of disease research on the basis of existing experience. The recent burst reference reflects research on testing the effectiveness and development prospects of fNIRS by measuring the PFC activity in Parkinson’s disease patients during dual-task walking, which was published by Nieuwhof et al. (2016).

### 4.3. Limitations

Although we performed a comprehensive, objective, and visual analysis of publications related to the use of fNIRS in clinical disease research and the relevant developing trends, this study still had some limitations. First, the topic search was only conducted in WoS and did not include other databases, such as China National Knowledge Infrastructure (CNKI). Second, bibliometric software cannot distinguish the abbreviation of the terms with different names, potentially causing deviations in the statistical results.

## 5. Conclusion

In conclusion, this study revealed the status of relevant studies, important topics, and trends related to the use of fNIRS in clinical disease research from 2011 to 2022. Over the past decade, the number of relevant articles has grown significantly. In addition, scholars can use fNIRS technology to conduct more relevant neuroscience research in children and adolescents. Notably, AD is another hot topic in this field. As a relatively new neurological imaging technique, fNIRS has developed rapidly in the field of disease diagnosis. Simultaneously, using the advantages of fNIRS, researchers can strengthen the combination of fNIRS with traditional imaging technology.

## Author contributions

XYe, RS, and FL conceptualized the study. LH, XYa, YZ, and YS collected the data. XYe, LP, and NS analyzed the data and drafted the manuscript. XYe, NS, JX, RS, and FL revised the final version of the manuscript. All authors contributed to the article and approved the submitted version.
